# Integrated propranolol, methylprednisolone, and surgery in managing a rare case of infantile hemangioma with concurrent cleft lip and palate

**DOI:** 10.1016/j.amsu.2020.06.015

**Published:** 2020-06-19

**Authors:** Ishandono Dachlan, Siti Isya Wahdini, Indri Lakhsmi Putri, Muhammad Rosadi Seswandhana, Aditya Wicaksana, Aditya Rifqi Fauzi

**Affiliations:** a Plastic, Reconstructive, and Aesthetic Surgery Division, Department of Surgery, Faculty of Medicine, Universitas Gadjah Mada/Dr. Sardjito Hospital, Yogyakarta, 55281, Indonesia; b Plastic, Reconstructive, and Aesthetic Surgery Division, Department of Surgery, Faculty of Medicine, Universitas Airlangga/Airlangga University Hospital, Surabaya, Indonesia

**Keywords:** Hemangioma, Cleft lip and palate, Propranolol, Methylprednisolone, Rare case

## Abstract

Infantile hemangioma (IH) with concurrent cleft lip and palate is a rare case. Surgical management is often considered as the best management for infantile hemangioma with concurrent cleft lip and palate. However, considering the functionality aspect and aesthetic appearance, a plastic surgeon can also consider non-surgical management without interrupting the surgical timeline for the cleft lip and palate. This case report aimed to describe the role of oral propranolol and oral methylprednisolone for infantile hemangioma with concurrent cleft lip and palate alongside the surgical management for cleft lip and palate.

A 2-month-old presented with complaints of swelling in her right upper nose and cheek along with cleft lip and palate. She was treated with oral propranolol and oral methylprednisolone. Labioplasty was performed when she was three months old. Palatoplasty and nasorraphy were done when she was one year old. A significant reduction of the hemangioma was seen and the corrective procedures showed a good result.

The use of propranolol and methylprednisolone for infantile hemangioma in our patient shows a good result even when combined with labioplasty, palatoplasty, and nasorraphy for cleft lip and palate.

The management of infantile hemangioma with concurrent cleft lip and palate using oral propranolol and oral methylprednisolone shows a good result with no side effects and can be elaborated with labioplasty, palatoplasty, and nasorraphy, and will not interrupt the cleft lip and palate surgical timeline.

## Highlights

• Propranolol and methylprednisolone orally provide an effective and safe modality of treatment.
• Can be elaborated with surgery for cleft lip and palate, thus shows a good result without interrupting the surgical timeline.
• Satisfying result was observed.

## 1 Introduction

Infantile hemangiomas (IH) are one of the most common benign vascular malformations in children, with a prevalence of 5–10% of cases in infants [1]. Hemangiomas usually develop in the infancy period, with an increase incidence rate in female Caucasians [2,3]. The pathogenesis pathway of this abnormality hypothesizes it results from aberrant proliferation and differentiation of a hemogenic endothelium having a neural crest phenotype and a capacity for endothelial, hematopoietic, mesenchymal, and neuronal differentiation [4,5]. IH can be found on the head and face, extremities, and other regions of the body. Uncomplicated hemangioma can resolve spontaneously by the age of 5 years, and nearly 50% of simple hemangioma become involuted [6].

There are many clinical therapies for IH patients, which can be used such as surgical excision, lasers, corticosteroid injections, embolization, cryosurgery, radiotherapy, and cyclophosphamide [6]. Up till now, the most commonly used medication is oral prednisolone. Propranolol is considered safe when administered in appropriate patients and can also be used to treat IH. Oral prednisolone and propranolol showed excellent results individually for IH treatment [7]. The combination of these two drugs significantly reduces tumor size in the proliferation phase and improves tumor color with minimal incidence of mild side effects. Children are proven to have high tolerance with this therapeutic method, and the combination treatment is proven to be highly safe and has made a significant difference to clinical practice [8].

Cleft lip is also a common condition in children. Cleft lip incidence varies between ethnicity, with 0.41:1000 in African Americans, 1:1000 in Caucasians, and 2.1:1000 in Asians [6]. However, the condition of hemangiomas accompanied by cleft lip is a rare case [9].

This report aims to describe the combination role of propranolol and methylprednisolone in the case of hemangioma accompanied by surgical therapy in an infant with cleft lip and palate. This case report has been reported in line with the SCARE 2018 criteria [10].

## 2 Presentation of case

**Fig. 1:**
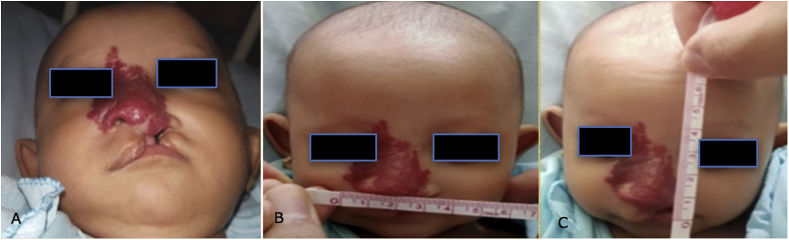
Propranolol Day-1/one month before labioplasty.

**Fig. 2:**
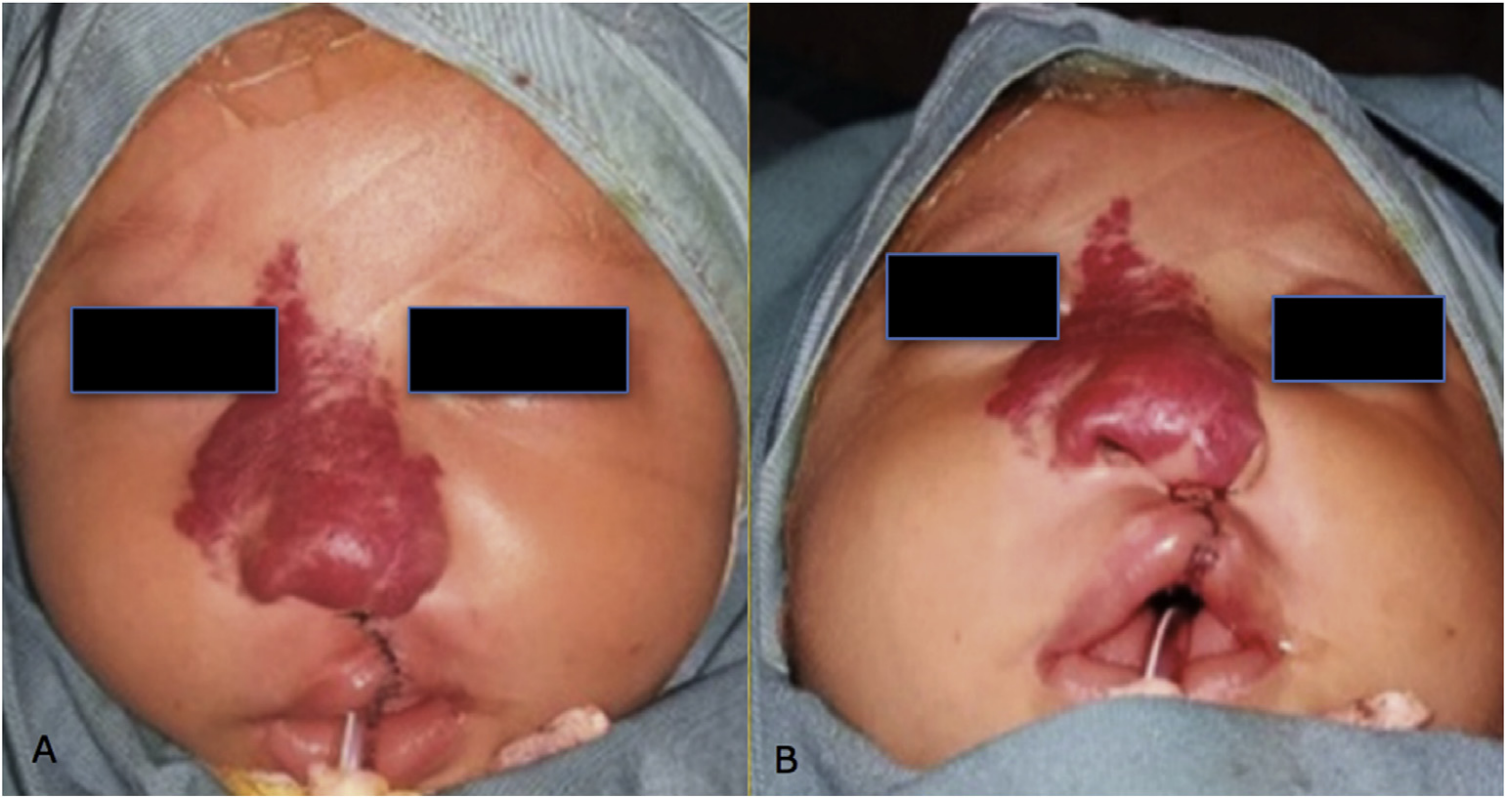
Post-labioplasty, after taking propranolol for one month.

A 2-month-old girl was brought to our hospital complaining of swelling in her right upper nose and cheek. Physical examination revealed hemangioma in the nasal and malar region with a size of 35 mm × 32 mm along with cleft lip and palate (-SHAL) as can be seen in Fig. 1. She was treated with oral propranolol and oral methylprednisolone since then. The initial dose for oral propranolol was 1 mg/kg/day in three divided doses, and the initial dose for oral methylprednisolone was 2 mg/kg/day in two divided doses. The dose for propranolol was increased 0.5 mg/kg/day until reaching 2 mg/kg/day and maintained for one year period, and the dose for methylprednisolone was gradually reduced to zero for six weeks. Labioplasty was performed when she was three months old and the results after taking propranolol for 1 month can be seen in Fig. 2. Palatoplasty and nasorraphy were done when she was one year old.

**Fig. 3:**
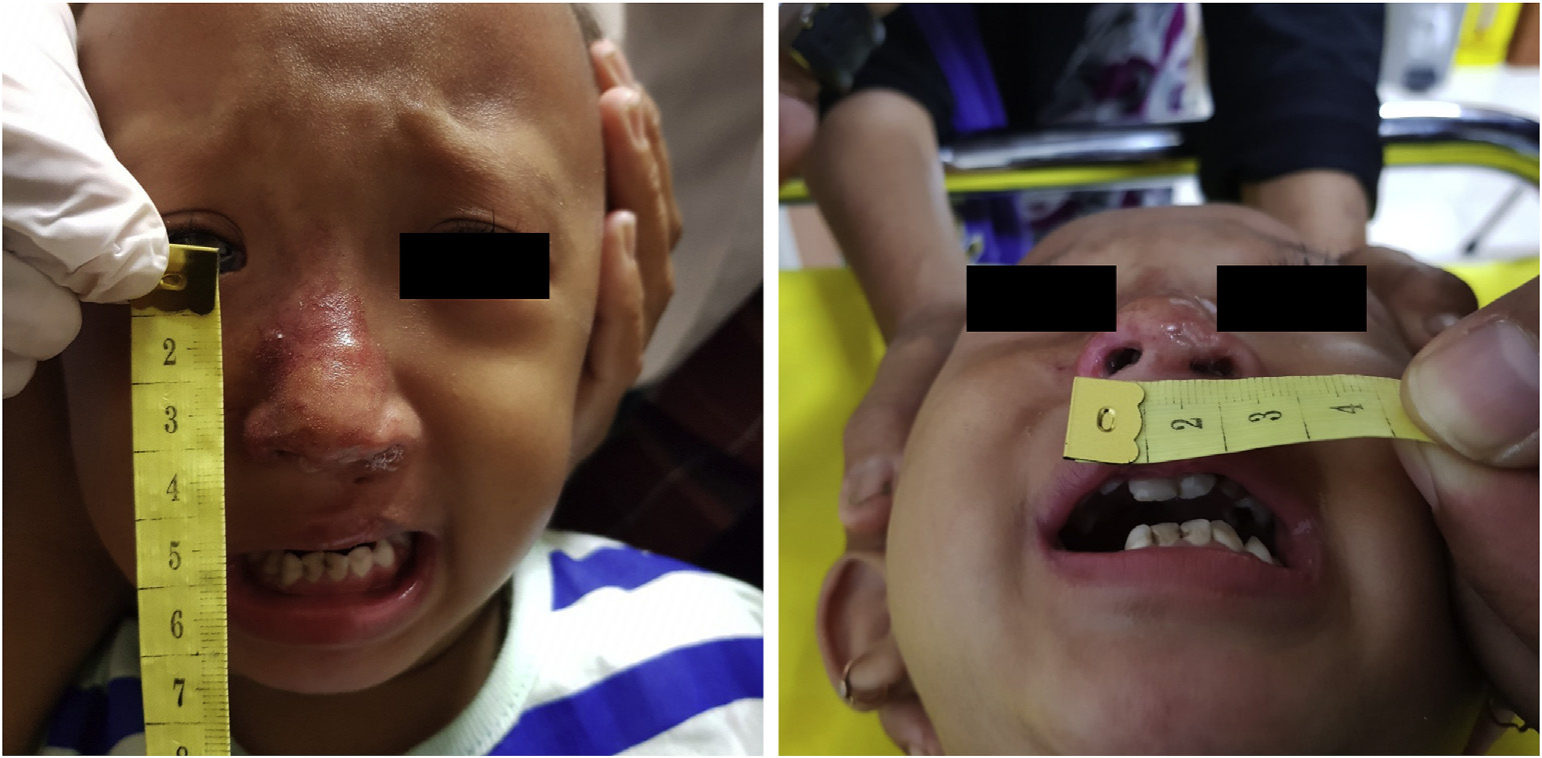
Post-palatoplasty and nasorraphy, after taking propranolol for one year.

There was a significant reduction of the hemangioma in the nasal region, leaving a little mark in the malar region as can be seen in Fig. 3. Labioplasty, palatoplasty, and nasorraphy procedures showed a good result. The patient's family (especially her mother) was happy and satisfied with the results.

## 3 Discussion

IH with concurrent cleft lip and palate is a rare case and possesses a particular challenge for clinicians. The role of propranolol to accelerate the involution phase of hemangioma was discovered unexpectedly by Léauté-Labrèze et al. [11], when they initiated propranolol for infants with obstructive hypertrophic cardiomyopathy with hemangioma. Propranolol is a non-selective β1‐ and β2‐adrenoceptor antagonist which causes vasoconstriction, impeding the proangiogenic signals, and stimulating apoptosis in endothelial cells’ proliferation in regards to IH [12]. Still, the potential side effects of propranolol such as hypotension, bradycardia, and hypoglycemia, should also be monitored.

Prior to the use of propranolol as IH treatment, corticosteroids were considered as the drug of choice for hemangioma. Corticosteroids were also found to be an effective treatment for thrombocytopenia in a child with hemangioma and prevented complications such as hemorrhagic events [13]. However, long-term use of corticosteroids has several side effects such as hypertension, glucose intolerance, wound complications, increased risk of infections, etc. Nonetheless, corticosteroids are still included in the treatment of IH. The exact method for reducing corticosteroid doses in IH patients is unknown. From the previous study, adequate time for treatment is around 6–8 weeks [14]. In addition to the therapeutic period, propranolol administration for IH was recommended for a duration of 6 months and up to 12 months [15].

Until now, hemangioma management is still controversial. Traditionally, observation has been a therapeutic option in the hope that the lesions disappear spontaneously. This choice arises when surgical excision or other therapies may worsen the condition. However, with changing techniques, surgery can be the first-choice therapy, especially in the location of lesions with significant cosmetic or functional defects [16]. Unfortunately, after we gave a choice for surgery or conservative therapy, the parents of the patient preferred conservative treatment.

However, in our case, we did not observe any side effects of both propranolol and methylprednisolone in our patient. Moreover, the use of propranolol and methylprednisolone for IH in our patient shows a good result even when combined with labioplasty, palatoplasty, and nasorraphy for cleft lip and palate.

Thus, our case report suggests that propranolol and methylprednisolone are safe and effective in the management of IH and can be elaborated without interrupting a cleft lip and palate surgical timeline.

## 4 Conclusions

The management of IH with concurrent cleft lip and palate using oral methylprednisolone and oral propranolol shows a good result with no side effects and can be elaborated with labioplasty, palatoplasty, and nasorraphy, and will not interrupt the cleft lip and palate surgical timeline.

## Funding

The authors declare that this study had no funding source.

## Ethical approval

The informed consent form was declared that patient data or samples will be used for educational or research purposes. Our institutional review board also do not provide an ethical approval in the form of case report.

## Consent

Written informed consent was obtained from the parent of the patient for publication of this case report and accompanying images. A copy of the written consent is available for review by the Editor-in-Chief of this journal on request.

## Registration of research studies

This is not a ‘first in humans’ report, so it is not in need of registration.

## Guarantor

Ishandono Dachlan.

## Provenance and peer review

Not commissioned, externally peer reviewed.

## CRediT authorship contribution statement

**Ishandono Dachlan:** Conceptualization. **Siti Isya Wahdini:** Data curation, Resources, Investigation. **Indri Lakhsmi Putri:** Data curation, Resources, Investigation. **Muhammad Rosadi Seswandhana:** Validation. **Aditya Wicaksana:** Writing - original draft. **Aditya Rifqi Fauzi:** Writing - review & editing.

## Declaration of competing interest

No potential conflict of interest relevant to this article was reported.
